# Anisotropy of Local Stress Tensor Leads to Line Tension

**DOI:** 10.1038/srep09491

**Published:** 2015-04-02

**Authors:** Mingzhe Shao, Jianjun Wang, Xin Zhou

**Affiliations:** 1 Institute of Chemistry, Chinese Academy of Science, Beijing 100190; 2 School of Physics, University of Chinese Academy of Sciences, Beijing 100049

## Abstract

Line tension of three-phase contact lines is an important physical quantity in understanding many physical processes such as heterogeneous nucleation, soft lithography and behaviours in biomembrane, such as budding, fission and fusion. Although the concept of line tension was proposed as the excess free energy in three-phase coexistence regions a century ago, its microscopic origin is subtle and achieves long-term concerns. In this paper, we correlate line tension with anisotropy of diagonal components of stress tensor and give a general formula of line tension. By performing molecular dynamic simulations, we illustrate the formula proposed in Lennard-Jones gas/liquid/liquid and gas/liquid/solid systems, and find that the spatial distribution of line tension can be well revealed when the local distribution of stress tensor is considered.

Line tension is an important parameter for the interpretation of heterogeneous nucleation^1^, soft lithography^2^,^3^, and behaviours of biomembranes like budding, fission and fusion^4^,^5^,^6^. Although line tension is well-defined in thermodynamics by Gibbs long time ago, its existence, magnitude, sign, and some substantial issues, such as what leads to line tension, have long been discussing and studying in theories, simulations, and experiments^7^,^8^. The three-phase contact line (CL) is theoretically complicated because it involves at least three bulk phases and three interfaces. Thus investigation on line tension inevitably needs to discuss three-dimensional information such as the shape and structure of the three-phase contact zone^9^,^10^,^11^. Theoretical estimate of line tension is usually based on mean field theory^10^,^11^, involving density function theory (DFT)^12^, line tension can thus be related to intermolecular forces. A generally accepted theoretical estimate of line tension is on the order of 10^−12^ to 10^−10^ J/m^12^,^13^,^14^,^15^,^16^,^17^,^18^,^19^,^20^,^21^, while experiments under various conditions suggest a broader range varying from 10^−11^ to 10^−5^ J/m^7^,^22^,^23^,^24^,^25^. This mismatch cannot simply be ascribed to the simplicity of theoretical models and poor experimental techniques, the very different systems performed in these studies could also be a reason. As such more efforts are necessary in both theoretical/computational and experimental sides to have a complete picture of line tension.

Molecular dynamic (MD) simulation has directly brought a novel theoretical view of line tension from molecular interaction. Werder *et al.*^21^ brought the dependence of the contact angle on line tension with the “modified Young's equation”^7^,^24^ into the microscopic scale. They simulated water droplets of various sizes on graphite surfaces, the contact angle of the droplets was then measured to obtain the line tension in the order of 10^−10^ J/m. Another approach, “finite-size-scaling”, was also performed to extract line tension from free energy^26^,^27^,^35^. These methods usually require a series of simulations with various system sizes and data fitting. Moreover, line tension needs to be extracted from the dependence of the contact angle or surface tension on the size of system. On the other hand, surface tension can be easily related to molecular interactions. Kirkwood *et al.*^28^,^29^ provided a general method to study surface tension by a mechanical route using virial and stress tensor. The surface tension was calculated from the difference between the normal and tangential pressure of the surface, thus can be directly obtained from a single molecular simulation. This approach has been widely adopted in the calculation of surface tension^30^,^31^.

In this paper, following the similar spirit of relating surface tension to anisotropy of stress tensor^28^,^29^, we relate the total excess free energy of contact lines and interfaces as the deviation of the transverse pressure to the parallel pressure along the contact line. It is a direct approach to estimate line tension in a single simulation, and gives a general formula relating line tension to microscopic interaction. Based on the Irving-Kirkwood's definition of local stress tensor, we clearly show that the origin of line tension is the anisotropy of local stress tensor in the three-phase coexistence regions, similar to that of surface tension in interfaces.

## Theory

In a three-phase coexisting system, the differential of Helmholtz free energy is
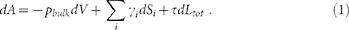
Here *p*_bulk_ is the pressure in the bulk region which is far away from any multiple-phase coexisting regions. It is defined as the free energy change while varying the volume of system, *V*, but keeping areas of interfaces, *S_i_*, and the total lengths of contact lines *L_tot_*, *i.e.*, 
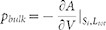
. Here *γ_i_* is surface tension of interface, and *τ* is line tension.

**Figure 1:**
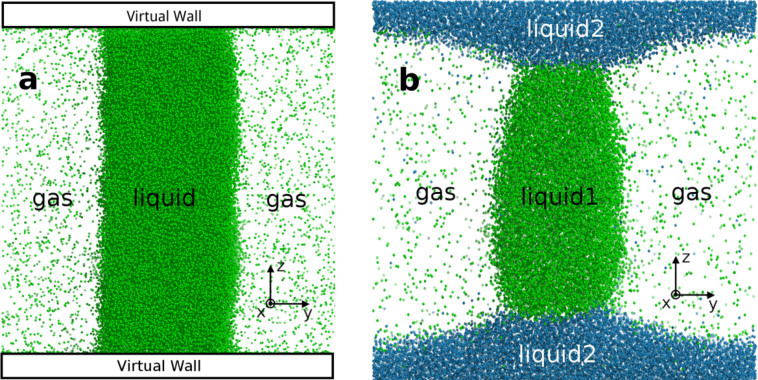
Two kind of three-phase coexisting system. (a) Virtual walls are built in perpendicular to *z*-direction, gas/liquid phase-separation occurs along *y*-direction, there are four contact lines at three-phase zones, which are parallel with *x*-axis. The flat gas/liquid surface is built to simplify our calculation, the contact angle is about 90 degrees by selecting parameters of adsorption potential of the walls. (b) A common gas/liquid/liquid three-phase coexisting system.

We consider a system having three phases, three interfaces and four contact lines as shown in Fig. 1, the contact lines are perpendicular to the paper plane, such as along *x*-axis, and the interfaces might be perpendicular to each other as shown in Fig. 1(a), or not, as Fig. 1(b). If varying the size of the transverse section of the system, *L_y_* → *L_y_ + dL_y_* and *L_z_* → *L_z_* + *dL_z_* while retaining the shape, *i.e.* setting 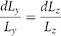, the area differential of any two-phase interface will be proportional to its area, 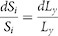, and will be independent of the curvature and the direction of normal vector of the interface. Thus, it can be shown that,
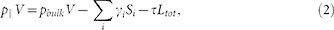

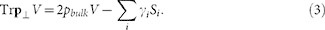
Here *p*_||_ = *p_xx_*, and 
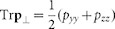
. The diagonal component of stress tensor is,
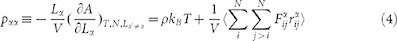
where *α* = *x*,*y*,*z* is a component of Cartesian coordinates. *L_α_* is the length of box in the *α* direction, and the volume *V* = *L_x_L_y_L_z_* for a cuboid box. 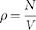 is density of the system, *k_B_* is the Boltzmann constant, and *T* temperature. 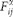 and 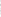 are the *α* component of force **F***_ij_* on the *j*th atom from the *i*th atom and distance vector **r***_ij_* = **r***_j_* − **r***_i_*, respectively. Angular bracket refers to the canonical ensemble average. Here we already suppose the molecular interaction is a pair additive force. The two terms^31^ are known as a kinetic term, arising from the change in momentum due to particles crossing the boundaries of an elemental volume, and a configurational term, related to the change in momentum due to intermolecular interactions between particles and possible external forces. It is worth noticing that the bulk pressure, *p_bulk_*, in multiple-phase coexistence systems may be different from any diagonal component of stress tensor, thus from both *p*_⊥_ and *p*_||_.

Equation (3) relates the total surface energy of the system to the difference of the bulk pressure from the perpendicular pressure, and is a generalized relation of surface tension in two-phase systems presented by Kirkwood and co-workers^28^, where surface tension is attributed to the difference between the normal and tangential pressure. We also have,
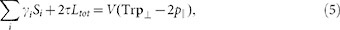
which is approximately equal to the total excess free energy of the multiple-phase coexisting regions, *A_ex_* = Σ*_i_γ_i_S_i_* + *τL_tot_*, if the total line tension *τL_tot_* is much smaller than the total surface energy. Here *L_tot_* = 4*L_x_* is the total length of contact lines. It is worthy to mention that the trace of the perpendicular stress Tr**p**_⊥_ can be calculated from any two orthogonal transverse directions in the plane.

From equation (2) and equation (3), we have one of primary results of this paper,
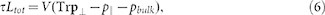
the line tension is attributed to the anisotropy of stress tensor, *i.e.*, the difference of the transverse stress from the tangential stress and bulk pressure.

To better understand the relation between line tension and anisotropy of stress, we define local stress tensor^31^,^32^ *P_αβ_*(**r**) at spatial position **r**, which is written as
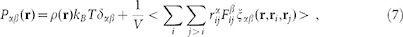
under the condition 
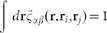
. Here *ρ*(**r**) is local density, *δ_αβ_* is Kronecker delta symbol. *ξ_αβ_* (**r**,**r***_i_*,**r***_j_*) is the fraction of the intermolecular virial between a given pair of molecules at **r***_i_* and **r***_j_* to be assigned to position **r**. It is easy to know the normal stress tensor *p_αβ_* is the average of the local quantities in the whole system. Irving-Kirkwood^29^ (IK) used
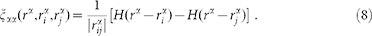
to get the local stress in their calculation of surface tension. Here *H*(*x*) is the Heaviside function. Another selection of the *ξ* function is also possible and not sensitive to the obtained local stress. In the paper, we follow IK's selection, perform equation (7) and equation (8) to attribute the virial of atom pairs into their spatial positions for the local stress.

In the calculation of line tension based on equation (6), we need to estimate the bulk pressure. There are many methods to do so. While **r** is far away from the interfaces and the contact lines, *P_αα_*(**r**) for any *α* gives the value of *p_bulk_*. In the interface regions, we may estimate the bulk pressure as Tr**P**_⊥_(**r**) − *P*_||_(**r**). In this paper, we calculate the average value of bulk pressure as
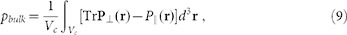
where *V_c_* refers to bulk or interface regions far away from the three-phase coexisting zones. Thus we could calculate the spatial distribution of line tension,
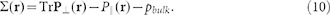
Σ(**r**) should be nonzero only in the three-phase contact regions, but zero in both interfacial regions and bulk regions. Thus, the total line tension can be integrated by limiting the integral region only in the three-phase contact regions without changing results. Similarly, we could have the local surface tension,
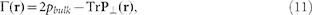
which is equivalent to the well-known formula Γ(**r**) = *P_n_*(**r**) − *P_t_*(**r**) in interfacial regions^28^,^29^, since *P_n_*(**r**) ≈ *p_bulk_*, and Tr**P**_⊥_ ≈ *p_n_* + *p_t_*. Here the index *n* and *t* mean the normal and tangential direction of interface, respectively.

It is worthy to point, while interfaces are not flat but curved, both equation (3) and equation (6) are correct, but the bulk pressure might be spatial inhomogeneous. Due to the Young-Laplace equation, the inner and outer pressure of a droplet are not equal. In that case, the bulk pressure shown in equation (3) and equation (6) is its average value over the whole system, which could be estimated by equation (9). In estimating the spatial distribution of line tension and surface tension from equation (10) and equation (11), the inhomogeneity of bulk pressure should be taken into account by using its local value to replace the average value in the two equations. In comparison with the spatial varying of line tension and surface tension, the inhomogeneity of bulk pressure is usually smaller and can be treated as background, thus the line tension and surface tension signal can be obtained. More details will be discussed below.

## Simulation details

We build two three-phase systems, one is a gas/liquid/solid system with a right contact angle between gas/liquid surface and solid wall to avoid curved interface, as shown in Fig. 1(a), and the other is a gas/liquid_1_/liquid_2_ system with curved interfaces and non-right contact angle, as shown in Fig. 1(b). The gas/liquid/wall system is equilibrated with, at most, 78109 argon atoms for 1 ns and run extra 4 ns for data collection. The argon is described by Lennard-Jones model with diameter *σ* = 0.336 nm, and the energy parameter *ε* is 0.9927 KJ·mol^−1^ corresponding to *k_B_T* with *T* = 119.4 *K*^33^. Two structureless walls are applied to represent the solid phase, the interaction between the wall and argon atoms is set as the similar Lennard-Jones function, the parameters are adjusted to make the gas/liquid interface be flat. Lennard-Jones potential of argon atoms is truncated and shifted spherically with cutoff radius *r_c_* = 1.2 nm. Periodic boundary conditions (PBC) are applied in only (*x*,*y*) while two parallel structureless solid walls are induced in *z*-direction. In the gas/liquid_1_/liquid_2_ system, liquid_1_ is argon and liquid_2_ is another Lennard-Jones fluid with *σ*_2_ = 0.3851 nm ≈ 1.15*σ*, and *ε*_2_ = 1.3188 KJ·mol^−1^ ≈ 1.33*ε*, the interact parameter between two kinds of particles is set as *σ*_12_ = 1.07*σ*, and *ε*_12_ = 0.57*ε*. This gas/liquid_1_/liquid_2_ system exhibits a more general phase contact zone. The simulations are performed at constant temperature *T* and volume *V*. A subcritical temperature is set to stabilize two planar gas/liquid interfaces. MD simulations are performed by using GROMACS version 4.5.5 to generate the molecular trajectories with a time step of 2 fs. The temperature is kept constant by using Berendsen thermostat^34^ with a time constant 0.2 ps.

## Results and Discussion

**Figure 2:**
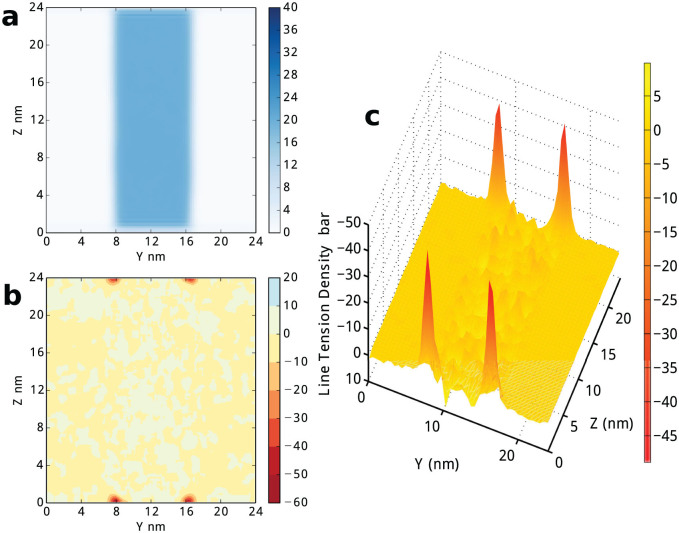
Spatial distribution of density and line tension in gas/liquid/wall model. The line tension distribution is generated by setting bin size as 0.2 *nm*. Interpolation is applied to generate smooth 3D graph. (a) the density distribution. (b) the line tension distribution contour plot. (c) the 3D plotting of the line tension distribution on (*y*,*z*) plane.

We first focus on the gas/liquid/solid three-phase system shown in Fig. 1(a). By equation (10), the spatial distribution of line tension in the system can be estimated, the result at *T* = 100 K is shown in Fig. 2, which clearly shows line tension only comes from the four three-phase contact zones. Here, we estimate the bulk pressure *p_bulk_* = 4.6 bar by equation (9) where the integration zone is set as 1/3 of the simulation box side length away from the four contact line regions. Indeed, Fig. 2(b) shows a background signal of the line tension around zero with fluctuation less than one colour scalebar (10 bar). There is no substantial difference between bulk fluid and the gas/liquid, gas/solid, and liquid/solid interfaces, even though the spatial distribution of particle density in Fig. 2(a) shows obviously the existence of these interfaces. The 3D plot in Fig. 2(c) present a direct view of the spatial distribution of the line tension density. Negative peaks of line tension density at contact lines are observed, their values are almost same and also at least five times larger than the background fluctuation. This phenomenon can be clearly elucidated with equation (6). In the homogeneous fluid zone, the line tension is zero, due to *p_bulk_* = *P_xx_*(**r**) = *P_yy_*(**r**) = *P_zz_*(**r**). While in the gas/liquid interface, since *p_bulk_* = *P_yy_*(**r**), *P_xx_*(**r**) = *P_zz_*(**r**), line tension is still zero. Similarly, In all the other interfaces, the line tension is also zero, except in the three-phase coexisting zones of the system. The result shows 4*τ* = −153 bar·nm^2^, the line tension in this system is −3.83 × 10^−12^ J/m, which is in agreement with previous results in both DFT calculation of Lennard-Jones system^12^ and experiments^18^,^22^.

**Figure 3:**
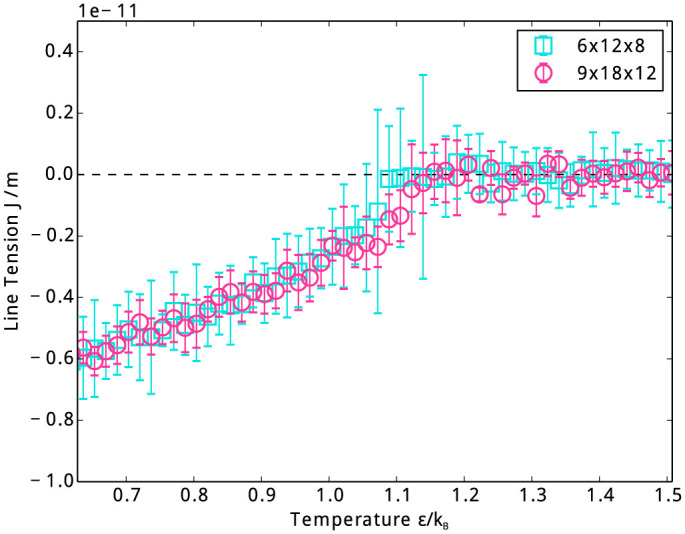
Temperature dependence of line tension. Two different sizes of simulation box are applied to detect the possible finite-size effects.

We further study the temperature dependence of line tension, the result is plotted in Fig. 3. In this system, when temperature is lower than the critical temperature *T_c_* ≈ 1.17*ε*/*k_B_*, the value of line tension is negative. Its absolute value is about 6 × 10^−12^ J/m at *T* = 0.67*ε*/*k_B_*, approximately linear change to zero as temperature rises to *T_c_*. That is, when temperature approaches *T_c_*, the gas/liquid interface disappears and so does the line tension. To detect the possible finite-size effects, we simulate two systems of different sizes, the obtained line tensions are equal within statistical errors.

A negative line tension is usually thought to make nucleation easier, as it decreases free energy barrier of nucleation. However, our obtained absolute value of line tension is relatively small, in the order of 10^−12^ to 10^−11^ J/m. This may only have influence in systems with molecular dimensions, while larger line tension could influence processes in the micrometer scale, such as the usual heterogeneous nucleation. Some other studies^12^,^17^,^18^,^19^,^22^,^25^ also show the same order of magnitude of line tension in similar systems. These results seem to imply that line tension is less likely to be crucial in nucleation at least in the present system.

**Figure 4:**
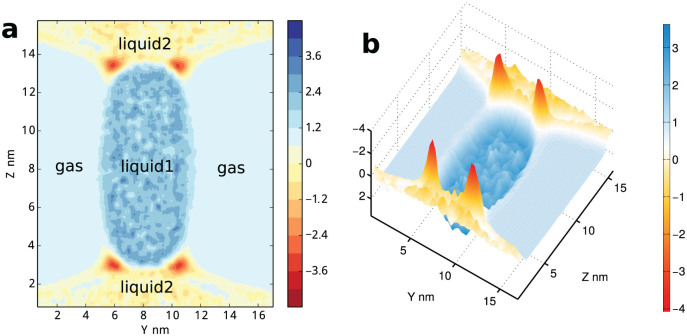
Spatial distribution of *P_yy_*(r) + *P_zz_*(r) − *P_xx_*(r) in gas/liquid_1_/liquid_2_ model. It corresponds to line tension but containing bulk pressure as background. The anisotropy of stress is rescaled with the average stress *p_yy_* + *p_zz_* − *p_xx_* ≈ 5.1 bar as unit.

**Figure 5:**
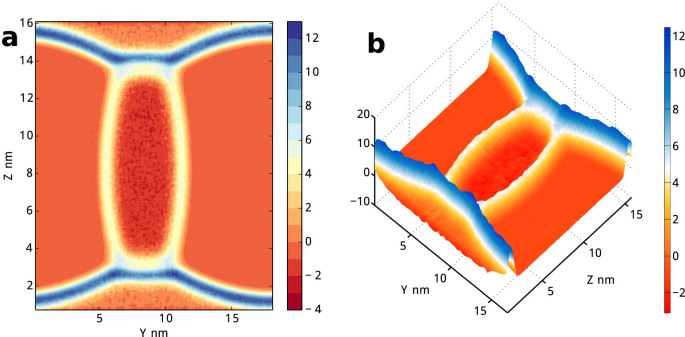
Spatial distribution of −(*P_yy_*(r) + *P_zz_*(r)) in gas/liquid_1_/liquid_2_ model. It corresponds to surface tension but containing bulk pressure as background. The unit of stress in the figure is −(*p_yy_* + *p_zz_*) ≈ 12.1 bar.

To study more general cases, instead of using virtual wall, we perform simulations in a gas/liquid/liquid system, seeing Fig. 1(b). The details of local stress component in the *x*, *y* and *z*-direction are shown in Supplementary, Fig. S1–S6. *P_xx_*(**r**) decreases on gas/liquid_1_, gas/liquid_2_ and liquid_1_/liquid_2_ surfaces because all these surfaces are parallel to the *x*-axis. *P_yy_*(**r**) has substantial decrease on gas/liquid_1_ and gas/liquid_2_ surfaces, but not on liquid_1_/liquid_2_ surfaces since liquid_1_/liquid_2_ is almost perpendicular to the *y*-axis. With the same reason, the *P_zz_*(**r**) has obvious decrease on liquid_1_/liquid_2_ surfaces. All stress components have slightly larger values in liquid_1_ than in gas phase. The gas pressure is around 5 bar, while liquid_1_ 11 bar. This could be explained with the well-known Young-Laplace equation 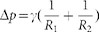, with the obtained gas/liquid surface tension *γ* ~ 80 bar·nm, and the two principle curvature radius *R_1_* ≈ 15 nm, and 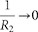. The pressure in liquid_2_ is smaller than that in gas, due to the negative curvature of the liquid_1_/liquid_2_ and gas/liquid_2_ interfaces. In addition, in the interface between two liquids, the tangential components of local stress show a double-layer structure (seeing Fig. S1, S2 and S6), indicates a complicate coexisting structure in the atomic scale. In the system, we can still estimate the average bulk pressure by using equation (9) and calculate the line tension from equation (6). But we can not simply use the average bulk pressure to extract the spatial distribution of line tension density, since the bulk pressure in gas and liquids differs at this scale. We plot *P_yy_*(**r**) + *P_zz_*(**r**) − *P_xx_*(**r**) in Fig. 4 (using its average value in the whole space, *p_yy_* + *p_zz_* − *p_xx_* ~ 5.09 bar as unit). The bulk pressure, although different in gas, liquid_1_ and liquid_2_ phases, is actually a smooth function of spatial position **r**, this corresponds to the background of the signal. Thus the line tension density distribution is nonzero only in the three-phase contact regions. We also calculate the surface tension density distribution by using equation (3). We similarly plot −(*P_yy_*(**r**) + *P_zz_*(**r**)) in Fig. 5 (with −(*p_yy_* + *p_zz_*) ~ 12.12 bar as unit). In this case, the bulk pressure as background is almost ignorable in comparison with the surface tension signal.

**Figure 6:**
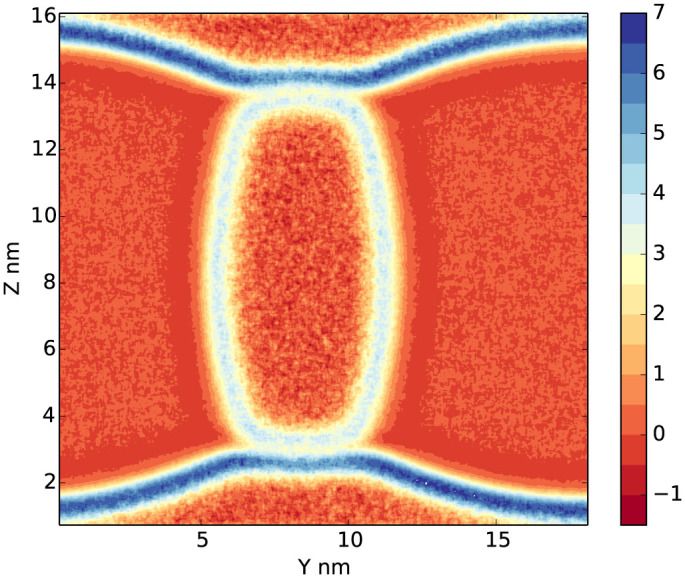
Spatial distribution of *P_yy_*(r) + *P_zz_*(r) − 2*P_xx_*(r) in gas/liquid_1_/liquid_2_ model. It approximately gives the total excess free energy distribution due to the multiple-phase coexistence. The unit of stress in the figure is *p_yy_* + *p_zz_* − 2*p_xx_* ≈ 22.3 bar.

With equation (5), we are aware of the total excess free energy due to the multiple-phase coexistence *A_ex_* = Σ*_i_γ_i_S_i_* + *τ L_tot_* approximately equals *V*(Tr**p**_⊥_ − 2*p*_||_) since *τL_tot_* is sufficiently small. Thus, the value of free energy per unit volume is about 22 bar. As shown in Fig. 6, its spatial distribution in (*y*,*z*) plane is quite similar with surface tension density distribution in Fig. 5. In the figure, even times by two, the line tension is still inconspicuous in the distribution map. The surface tension density on interfaces is significant, and can be read directly. They are approximately 130 bar, 100 bar and 80 bar for gas/liquid_2_, liquid_1_/liquid_2_ and gas/liquid_1_ interface respectively. The line tension density is mingled with surface tension influence in this figure, its effect is relatively inconspicuous in this system when compared with surface tension. After all, the bulk zone of gas, liquid_1_ and liquid_2_ neither contributes to surface tension, nor to line tension. It is specially worthy to mention, we found that the interfaces between two liquids show a slight double-layer structure, corresponding more complicate liquid-liquid interfaces in atomic scale than liquid-gas interfaces. More details about the double-layer interfaces are shown in Supplementary. The atom-scale layering^36^ between two concentrated phases usually exists, such as in our gas/liquid/wall system (data not shown). In the cases, MD simulations could provide detailed pictures about the anisotropy of local stress tensor. More analyses, such as possible comparison with DFT are very interesting, and will be performed in future works.

## Conclusion

As a summary, this present paper contains a relatively systematic account of the investigations of three-phase contact line tension. A statistical mechanics formula is developed to estimate line tension by a single MD simulation. Similar to the widely accepted method for the calculation of surface tension, where the difference between normal and tangential pressure components is used to extract surface tension, the line tension is calculated from the difference of the normal pressure to the tangential pressure and to the bulk pressure. We verified this method in two three-phase coexisting systems. Neither interfaces, nor bulk regions contribute to the line tension, only the anisotropy of local stress tensor in the three-phase contact regions leads to line tension. The general relation between line tension and anisotropy of stress tensor is in agreement with the result in a recent work^12^, where DFT was applied and the anisotropy of attractive force in the coexistence regions was found to mainly contribute to line tension. The obtained sign (negative) and magnitude (10^−11^ ~ 10^−12^ J/m) of line tension implicates that line tension might affect nucleation only when the size of nucleus is at molecular scale.

## Author Contributions

J.W. and X.Z. designed the project. M.S. and X.Z. performed the project. M.S., J.W. and X.Z. wrote and reviewed the manuscript.

## Supplementary Material

Supplementary Information Supplementary information

## References

[b1] Auer S. & Frenkel D. Line tension controls wall-induced crystal nucleation in hard-sphere colloids. Phys. Rev. Lett. 91, 1 (2003). 10.1103/PhysRevLett.91.015703 12906553

[b2] Peters R. D., Yang X. M., Kim T. K. & Nealey F. Wetting behavior of block copolymers on self-assembled films of alkylchlorosiloxanes: effect of grafting density. Langmuir 16, 9620 (2000).

[b3] Lopes W. A. & Jaeger H. M. Hierarchical self-assembly of metal nanostructures on diblock copolymer scaffolds. Nature 414, 735 (2001). 11742395 10.1038/414735a

[b4] Lipowsky R. Budding of membranes induced by intramembrane domains. J. Phys. II France 2, 1825 (1992).

[b5] Baumgart T., Hess S. T. & Webb W. W. Imaging coexisting fluid domains in biomembrane models coupling curvature and line tension. Nature, 425, 821 (2003) 14574408 10.1038/nature02013

[b6] Sriram I. & Schwartz D. K. Line tension between coexisting phases in monolayers and bilayers of amphiphilic molecules. Surf. Sci. Rep. 67, 143 (2012).

[b7] Amirfazli A. & Neumann A. W. Status of the three-phase line tension. Adv. Coll. Inter. Sci. 110, 121–141 (2004). 10.1016/j.cis.2004.05.001 15328061

[b8] Schimmele L., Napiórkowski M. & Dietrich S. Conceptual aspects of line tensions. J. Chem. Phys. 16, 164715 (2007). 17979379 10.1063/1.2799990

[b9] Jerison E., Xu Y., Wilen L. & Dufresne E. Deformation of an elastic substrate by a three-phase contact line. Phys. Rev. Lett. 106, 186103 (2011). 21635105 10.1103/PhysRevLett.106.186103

[b10] Widom B. Line Tension and the shape of a sessile drop. J. Phys. Chem. 99, 2803–2806 (1995).

[b11] Indekeu J. O., Koga K. & Widom B. How much does the core structure of a three-phase contact line contribute to the line tension near a wetting transition? J. Phys. Cond. Matt. 23, 194101 (2011). 10.1088/0953-8984/23/19/194101 21525549

[b12] Weijs J. H., Marchand A., Andreotti B., Lohse D. & Snoeijer J. H. Origin of line tension for a Lennard-Jones nanodroplet. Phys. Fluids 23, 022001 (2011).

[b13] Getta T. & Dietrich S. Line tension between fluid phases and a substrate. Phys. Rev. E 57, 655 (1998).

[b14] Solomentsev Y. & White L. Microscopic drop profiles and the origins of line tension. J. Colloid Interface Sci. 218, 122 (1999). 10489286 10.1006/jcis.1999.6389

[b15] Derjaguin B. V. & Gutop Y. V. Disjoining pressure and equilibrium of free films. Colloid J. USSR 27, 574 (1965).

[b16] Winter D., Virnau P. & Binder K. Monte carlo test of the classical theory for heterogeneous nucleation barriers. Phys. Rev. Lett. 103, 225703 (2009). 20366110 10.1103/PhysRevLett.103.225703

[b17] Quéré D. Surface wetting: Model droplets. Nature Mater. 3, 79 (2004). 14755259 10.1038/nmat1062

[b18] de Feijter J. A. & Vrij A. II. Smallest stable radius of a circular liquid film. Electrochem. 37, 39 (1972).

[b19] Scocchi G., Sergi D., D'Angelo C. & Ortona A. Wetting and contact-line effects for spherical and cylindrical droplets on graphene layers: A comparative molecular-dynamics investigation. Phys. Rev. E 84, 1 (2011). 10.1103/PhysRevE.84.061602 22304097

[b20] Liu Y., Wang J. & Zhang X. Accurate determination of the vapor-liquid/solid contact line tension and the viability of Young equation. Sci. Rep. 3, 2008 (2013). 23774479 10.1038/srep02008 PMC3684806

[b21] Werder T., Walther J. H., Jaffe R. L., Halicioglu T. & Koumoutsakos P. On the Water-Carbon Interaction for Use in Molecular Dynamics Simulations of Graphite and Carbon Nanotubes. J. Phys. Chem. B 107, 1345 (2003).

[b22] Heim L. O. & Bonaccurso E. Measurement of line tension on droplets in the submicrometer range. Langmuir 29, 14147 (2013). 24156499 10.1021/la402932y

[b23] Drelich J., Wilbur J. L., Miller J. D. & Whitesides G. M. Contact angles for liquid drops at a model heterogeneous surface consisting of alternating and parallel hydrophobic/hydrophilic strips. Langmuir 12, 1913 (1996).

[b24] Li D. & Neumann A. W. Determination of line tension from the drop size dependence of contact angles. Colloids Surf. 43, 195 (1990).

[b25] Guillemot L., Biben T., Galarneau A., Vigier G. & Charlaix É. Activated drying in hydrophobic nanopores and the line tension of water. Proc. Natl. Acad. Sci. U.S.A. 109, 19557 (2012). 23144219 10.1073/pnas.1207658109 PMC3511739

[b26] Binder K., Block B., Das S. K., Virnau P. & Winter D. Monte carlo methods for estimating interfacial free energies and line tensions. J. Stat. Phys. 144, 690 (2011).

[b27] Binder K. Monte Carlo calculation of the surface tension for two- and three-dimensional lattice-gas models. Phys. Rev. A 25, 1699 (1982).

[b28] Kirkwood J. G. & Buff F. P. The statistical mechanical theory of surface tension. J. Chem. Phys. 17, 338 (1949).

[b29] Irving J. H. & Kirkwood J. G. The statistical mechanical theory of transport processes. IV. The equations of hydrodynamics. J. Chem. Phys. 18, 817 (1950).

[b30] Ghoufi A., Goujon F., Lachet V. & Malfreyt P. Expressions for local contributions to the surface tension from the virial route. Phys. Rev. E 77, 031601 (2008). 10.1103/PhysRevE.77.031601 18517389

[b31] Gloor G. J., Jackson G., Blas F. J. & De Miguel E. Test-area simulation method for the direct determination of the interfacial tension of systems with continuous or discontinuous potentials. J. Chem. Phys. 123, 134703 (2005). 16223322 10.1063/1.2038827

[b32] Schofield P. & Henderson J. R. Statistical mechanics of inhomogeneous fluids. Proc. R. Soc. A 379, 231 (1982).

[b33] Zhou D., Zeng M., Mi J. & Zhong C. Theoretical study of phase transition, surface tension, and nucleation rate predictions for argon. J. Phys. Chem. B 115, 57 (2011). 21162588 10.1021/jp104969c

[b34] Berendsen H. J. C., Postma J. P. M., DiNola A. & Haak J. R. Molecular dynamics with coupling to an external bath. J. Chem. Phys. 81, 3684 (1984).

[b35] Tröster A., Oettel M., Block B., Virnau P. & Binder K. Numerical approaches to determine the interface tension of curved interfaces from free energy calculations. J. Chem. Phys. 136, 064709 (2012). 22360217 10.1063/1.3685221

[b36] Ingebrigtsen T. & Toxvaerd S. Contact Angles of Lennard-Jones Liquids and Droplets on Planar Surfaces. J. Phys. Chem. C, 111, 8518–8523. (2007).

