# Supplementary material for: Anisotropy of Local Stress Tensor Leads to Line Tension
Source: Sci Rep. 2015 Apr 2;5:9491. doi: 10.1038/srep09491 (PMC4382629; doi:10.1038/srep09491)
Supplement: Supplementary Information [file srep09491-s1.pdf]

Supplementary of
Anisotropy of Local Stress Tensor Leads to Line Tension

Mingzhe Shao,Jianjun Wang
Institute of Chemistry, Chinese Academy of Science, Beijing 100190
wangj220@iccas.ac.cn

Xin Zhou
School of Physics, University of Chinese Academy of Sciences, Beijing 100049
xzhou@ucas.ac.cn

We provide detailed simulation data of gas-liquid1-liquid2 system here.

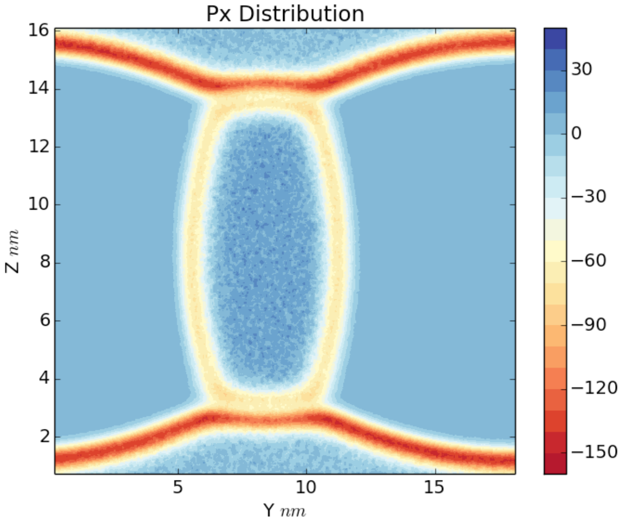

Fig. S1: p_x on (y,z) plane. The substantial decrease of p_x occurs on gas-liquid1, gas-liquid2 and liquid1-liquid2 interfaces. p_x drops more on gas-liquid2 and liquid1-liquid2 surfaces than gas-liquid1 surfaces.

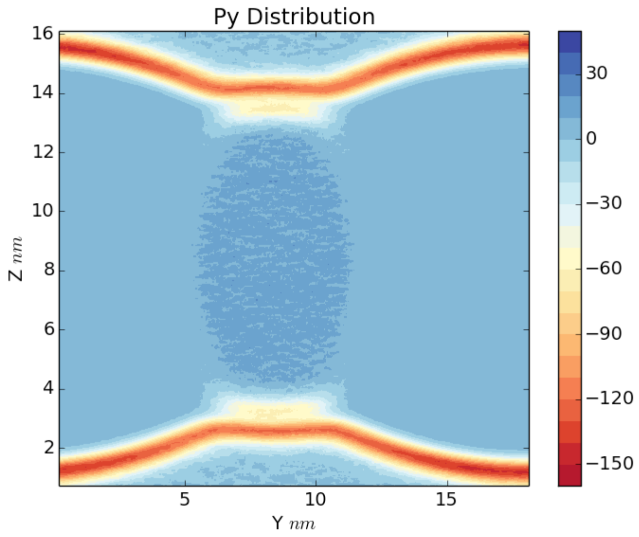

Fig. S2: p_y on (y,z) plane. The substantial decrease of p_y occurs on gas-liquid2 and liquid1-liquid2 interfaces for these interfaces are not perpendicular to y axis.

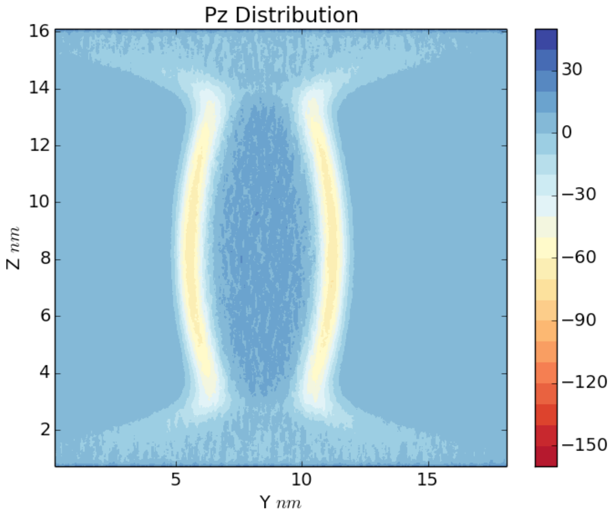

Fig. S3: p_z on (y,z) plane. The substantial decrease of p_z occurs on liquid1-liquid2 interfaces. There are pressure decreases on other interfaces since they are not completely perpendicular to z axis

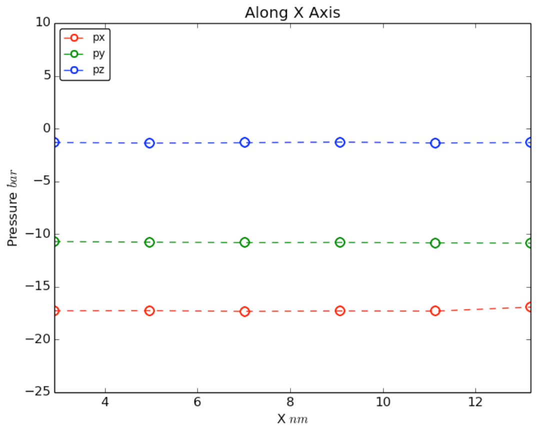

Fig. S4: pressure on x axis. The system is homogeneous in x direction. p_x decrease more than p_y, and p_z drops least. The average $p_y+p_z-2p_x \approx 22$ bar

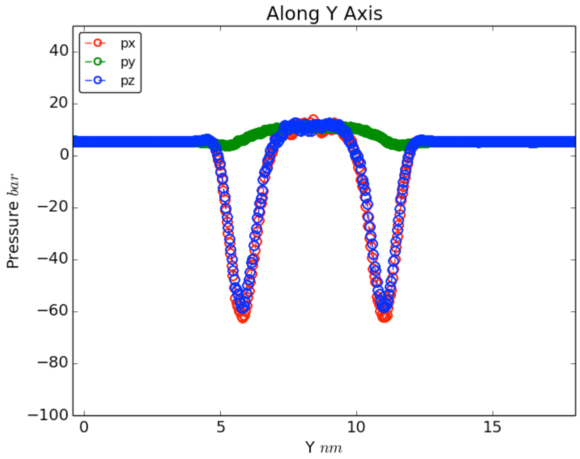

Fig. S5: pressure across the gas-liquid1 interfaces (along y axis). Only data far (1/4 box size) away from gas-liquid2 and liquid1-liquid2 interfaces are used to plot. The pressure is increasing in liquid1 bulk phase (caused by curved interfaces), and substantial pressure drop occurs on p_x and p_z.

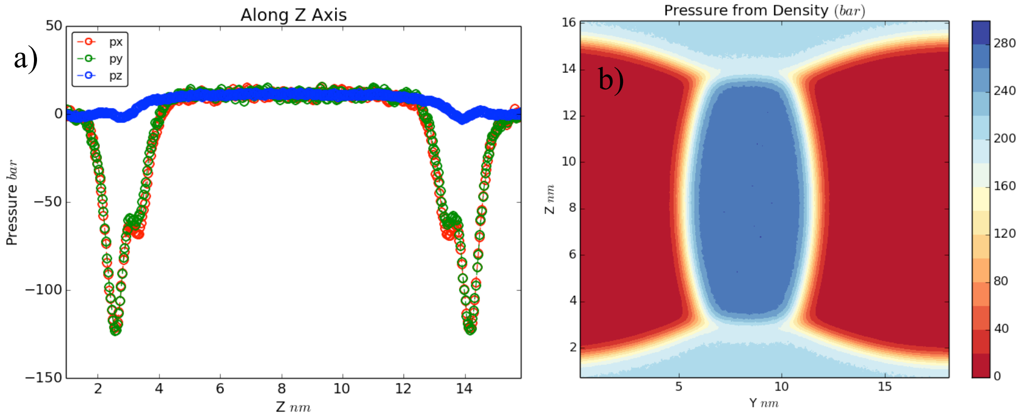

Fig. S6: a) pressure across the liquid1-lquid2 interfaces (along z axis). b) pressure from density. In (a), only data far (1 nm) away from gas-liquid1 and gas-liquid2 interfaces are used to plot. The pressure is increasing in liquid1 phase, and substantial pressure drop occurs on p_x and p_y. We may notice the tangential pressure decrease twice in each liquid1-liquid2 interface, and there seems to be two parts in each liquid1-liquid2 interface, this result is caused by a narrow density-decreasing layer in the liquid1-liquid2 interface, as shown in (b), the density is proportional to the pressure from density. This layer is a spontaneous phenomenon, it comes from the relatively small interact parameter ε_{12} we are using.
